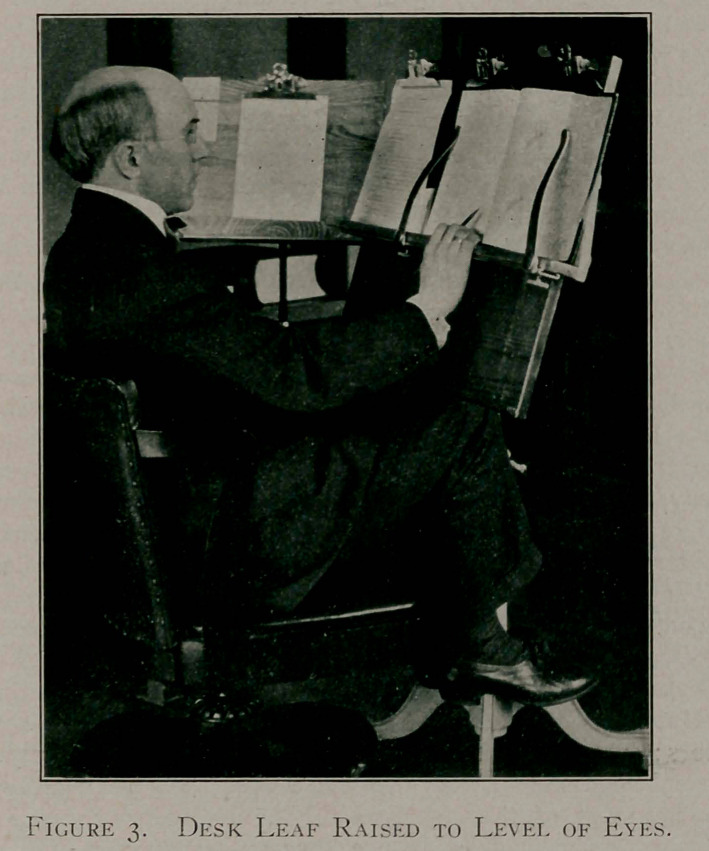# The Prevention and Cure of Disease by Means of the Inclined Desk-Leaf or Drawing Table

**Published:** 1910-08

**Authors:** George M. Gould

**Affiliations:** Ithaca, N. Y. Cayuga Heights


### The Prevention and Cure of Disease by Means of the Inclined Desk-leaf or Drawing Table

By GEORGE M. GOULD, M.D.
Ithaca, N. Y.

THE literary monks of the middle ages learned that writing on a flat table or desk was injurious to mental, ocular, and physical health. They habitually used one that was sharply pitched or inclined. We have still to profit by their experience, and when we come to a realising sense of the truth we shall then also recognise that its practical application will be of more value to the world in preventing and curing disease than, lumped together, all the medical and hygienic reforms of the day. Between four-fifths and nine-tenths of our school-educated young men and women have lateral curvature of the spinal column. And not a thing is done, not a finger lifted, either to prevent or to cure the disease. The morbid anomaly itself, together with its secondary or induced diseases, of the sixty or more millions of American citizens, could all have been prevented, or, if once in progress, could have been cured if set about early enough. The prevention could have been secured by means of the inclined desk-leaf in schools, and at home, used for all writing, and for most reading.

If one will make a simple test with the drawing-table here pictured the matter will become clear. Even a piece of board two feet long, inclined at an angle of 35°, placed upon an ordinary table, will illustrate. Write first upon the flat table and note the cramping, twisted, bent posture of the body and head, the laterally curved and strained vertebral column, the tilted and wrenched head, the skewed paper, the tilted shoulders,—all in order to secure the necessary clear vision, with the right eye, of the letters being written. Then place the paper straight up and down, opposite the right shoulder, upon a leaf inclined at about 35°, the head and body erect and squarely before the table. Why should we needlessly inflict the vast majority of the coming generation with deformity and disease? All the benefits of the public school system, do not counterbalance the evils it thoughtlessly begets in causing lateral curvature of the spinal columns of nine-tenths of the pupils. Of course the school boards, superintendents, teachers and taxpayers will not provide the inclined desks until the medical experts are convinced of the foregoing truths. But it is just as certain that the experts will not recognise and advocate the reform until intelligent parents and hygienists first arouse them. To these, therefore, the appeal must be made. "We must educate our masters."

I have long been urging parents, pupils, students, clerks, business men and literary workers to supply themselves with a simple cheap desk-leaf, inclined at an angle of about 35°, made of light one-half inch boards, to be placed upon the ordinary table or desk. Better still would be one of the many styles of drawing tables, folding trestles, and the like, manufactured by Messrs. Keuffel and Esser of New York. Two of the designs are pictured. There are many methods of construction possible whereby such devices might be supplied as cheaply as the present disease-breeding and deformity-producing school desks. I have word from a number of patients as to their relief from symptoms of weariness, irritation, and the like, by the use of such inclined tables. "Clips," "holders," springs, rubber bands, and the like, may be added to the outfit to hold books and papers, according to the special needs or tastes of the writers.

The inclined table has helped me to solve what, so far as I know, was a unique optico-medical problem.

Everlasting watchfulness for the peculiar ever-variant individualism of disease; tireless search for the causes and the nature of the individual condition; undoubted accuracy in measuring the degree of curability; unfailing ingenuity in devising and carrying out the methods of cure; and zealous sympathy, always,—these are demanded of the physician, but especially of the oculist. The great dangers are: routine,—reliance upon rules, the orderings of the text-books; trust in "laws;" obedience to prejudice: the superstition of "the typical case." In the last analysis there is no "typical case," and no set of "rules" that will apply mechanically even to two cases; every day will bring a patient whose disease is so nearly unique that the solution of the riddle must be sought from new starting points and resolved by new methods.

Among many illustrative cases of these truths worthy of being chronicled the following one is noteworthy:

Several months ago a gentleman, aged 41, occupying an important position at the head of a great institution, came to me giving a sad life-long history of eyestrain, and whose eyes them-

FIGURES 1 AND 2. DRAWING TABLES, SUBSTITUTES FOR SCHOOL DESKS.

selves showed the patent demonstrations of present disease. In the left eye the cornea was quite leucomatous and the iris was bound down to the capsule of the lens. In the right eye the cornea was also leucomatous, but part of the pupillary space was clear. Unfortunately the cloudy portion was the south-west, i. e., the lower and the outer two-thirds, the clear part being the north-east (upper and inner) third. The injury dated from infancy, since which time the man had been much afflicted with visual difficulties and resultant suffering. At the age of 11 glasses were ordered by a well-known oculist, and an iridectomy upon the left was performed. He had to leave school at 14. When about 16 years of age the internal and the external recti of the left eye were severed, for what purpose I could not learn or understand. His spectacles were changed thereafter "from time to time." In 1906 he had a sudden and severe attack of "neurasthenia," and was in the hospital for several months. In January, 1907, two able general physicians, in consultation, found "periodical blood-crises" "agoraphobia," and the like, and concluded that the inciting cause was eye-strain. Glasses were got from the oculist which destroyed all definition of objects beyond a few feet, and his health began to improve. The prescription was:

R. + S. O. 62 + C. 1.25 ax. 80° }<br>

L. + S. O. 62 + C. 1.25 ax. 80° } Distance

R. + S. 1 . 62 & cyl. }<br>

L. + S. O. 62 & cyl. } Near

During the last year the patient had suffered constantly from increasing pain in the eyes and head, and another oculist was consulted who at first ordered distance and near glasses. This order was:

R. + C. 1.50 ax. 75° }<br>

L. + S. 1.00 } Distance

R. + S. 1.00 & cyl. }<br>
L. + S. 1.00 } Near

The pain continuing as before, the left lenses were replaced by a "ground" or "steamed" lens designed to prevent vision, but the pain kept worsening.

After long and varied testing I secured 20|20 + vision with the right eye, but this degree of acuity was found by having the patient depress or tilt the head south-west by which maneuver the axis of macular vision could better pass through the clear space of the cornea. The final order was:

R. + S. 0. 25 + C. 1.25 ax. 70° }<br>

L. + S. 2. 00 + C. 4.00 ax. 105° } Distance

R. + S. 1. 25 & cyl, }<br>

L. Plano and Blinder } Near. Two separate pairs of spectacles

It is worthy of note that the symmetry of these astigmatic axes is a proof that the extreme amblyopia had not by any means been wholly caused by the leucoma. If he had always had a correct lens before this eye it would now have much better acuteness of vision. If the right eye was to be made useful for near vision, bifocals were out of the question because of the location of the bit of clear corneal space. The little vision possible to the left eye, even with its great increase by the high cylinder, was trebly necessary to keep the retina functional, to avoid dangers and accidents from the left side of the head and to preserve the possibility of some useful vision through operation in case of future loss of the right eye. It would have increased trouble to add + sph. 3.00 for near-work to this eye with its present accommodational paralysis, and the like, and a blinder was therefore ordered to exclude the left eye from near vision, which greatly aided in lessening confusion and eyestrain.

I had now concluded that if the man's work in life, and even his life itself, were to be continued, he must be made to see, in reading and writing, through the transparent one-third of corneal space at the north-east side. With this conviction the difficult problem was now given. How to solve it was certainly another matter! My first thought was to use for the right eye, a high-power prism, the apex north-east, at such an axis as would insure macular transfixion of the cone of rays with the least possible tilt of the head south-west. But the relative location of the corneal clear-space, the shapes of the nose and the orbital border disallowed the plan. The distance-lenses needed no study as the usual "flat" lenses provided sufficient means in walking and looking at distant objects. For reading and writing the right lens was ordered of large size and of toric shape, the optical center displaced north-east, and the whole fitted close under the orbital border and to the nasal bridge.

The patient had long been tormented by the appearance of great distortion of objects, but this at once disappeared upon getting the new spectacles.

It is evident that all this would not allow the patient to read and write as "needs must," in his case, if the book and writing paper should be held or placed, as with every one else, below a level of the eyes. The remaining problem was therefore to devise a method whereby the book and paper could be held above and to the left. I had for years been advising all patients to place their writing paper, books, etc., on a writing stand or leaf adjustable at any desired height and inclinable at an angle of about 35°. In this particular patient's case, it was only necessary to elevate the desk-leaf still higher, to the level of the eyes, and incline it at a still sharper angle. I show photographs of the device in use.

Soon after my patient got his adjustable stand, he made a discouraging and rather alarming report by long distance telephone. He recovered from his untoward symptoms in a few days, however, and went to work again. Soon there was a letter saying that he had been able to read an hour or two at a time, and had gone to the theater without ill effects.

Another letter followed two weeks later, from which I quote:

"Had faith in you from the first, but during two weeks preceding present week have had serious doubts of my own ability to fulfill your expectations. With present week there has certainly been a remarkable change and I am sure the improvement is not imaginary. Am using eyes regularly a small portion of each day and with complete comfort. Chief difficulty continues to be the artificial light at night and "stiff" or "lame" eyes in the morning. In addition to the tilted drawing table, I have the device reproduced in miniature for my desk. This second rack is at a fixed height above the desk and also at a fixed angle. With these two reading tables I find it very convenient to keep my work above my desk, with reference books and papers on the larger drawing table by my side."

The last report received was as follows:

"You told me that only Mrs. Eddy worked miracles. In this I believe you were wrong for I think we are having a little

FIGURE 3. DESK LEAF RAISED TO LEVEL OF EYES.

miracle of our own. My eyes have constantly improved and I am now using them from two to four and even five hours a day with the greatest comfort I have known for a long time. My chief trouble continues to be at night, although this too I believe to be growing less. I shall be glad to see you but cannot very well come to Ithaca unless you consider it necessary. I am still using the elevated desk with increasing satisfaction."

CAYUGA HEIGHTS.

## Figures and Tables

**Figures 1 and 2. f1:**
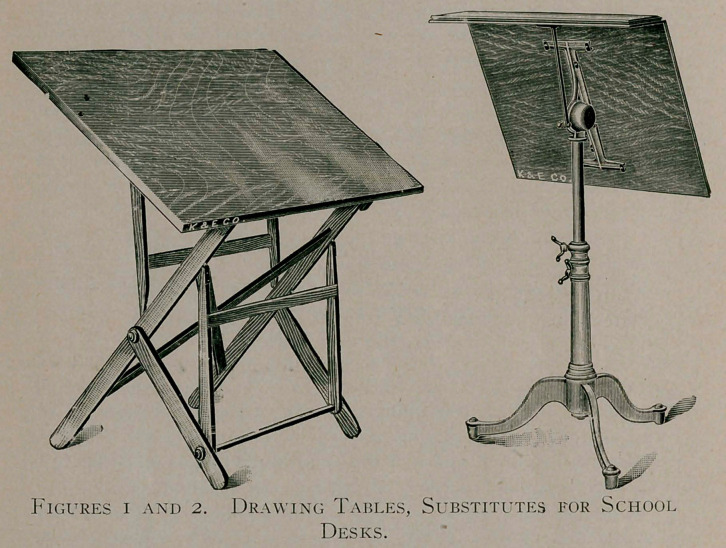


**Figure 3. f2:**